# When pursuing bad goals for good reasons makes it even worse: a social value approach to performance-avoidance goal pursuit

**DOI:** 10.1007/s11218-021-09623-0

**Published:** 2021-03-30

**Authors:** Wojciech Świątkowski, Benoît Dompnier

**Affiliations:** University of Lausanne, 1015 Lausanne, Switzerland

**Keywords:** Performance-avoidance goals, Social utility, Achievement, Goal complex

## Abstract

Consistently in achievement goal research, pursuing performance-avoidance goals has been associated with a decrease in achievement. Less is known to what extent this effect depends on the reasons underlying these goals’ endorsement. The present research uses a social value approach to assess how do performance-avoidance goals’ effects on achievement depend on the reasons anchored in social utility (goal endorsed in order to succeed) and in social desirability (goal endorsed in order to please one’s teachers). Based on five correlational samples meta-analyzed in Study 1, results showed that perceiving performance-avoidance goals as socially useful increased the negative effect of these goals on achievement. This moderating effect was replicated experimentally in Study 2. These findings support the relevance of studying achievement goal complexes and illustrate that performance-avoidance goals may lead to deleterious consequences even when endorsed for seemingly good reasons.

## Introduction

In the literature on achievement goals, performance-avoidance goals—aiming at not performing worse than others—are depicted as a maladaptive form of self-regulation. Decades of research have consistently demonstrated the deleterious impact these goals exert on many educational outcomes such as task interest or achievement (Hulleman et al. 2010). Late research has been devoted on understanding how different sorts of reasons underlying the pursuit of achievement goals change the consequences of their endorsement (e.g., Senko and Tropiano, 2016). The question that drew the attention among scholars in particular is whether the typical effects of achievement goals can be essentially accounted for by the reasons underlying the endorsement of these goals, or whether they result from the very combination between a goal and its corresponding reasons (i.e. the “goal-complex”; Elliot and Thrash, 2001; Sommet and Elliot, 2017; Vansteenkiste et al. 2010). Such an inquiry seems noteworthy with respect to performance-avoidance goals that are known to exert mostly negative effects on learning outcomes. Do these negative effects occur because individuals pursue them for some specific reasons? What would be the consequences of pursuing these goals had the underlying reasons been seemingly “good”, functional for one’s self-regulation?

The present research was conducted to address these queries using the social value approach to achievement goals (e.g., Dompnier et al. 2009). We investigated whether social utility and social desirability—two components of social value known to moderate the relationship between other types of achievement goals and achievement—could alter the link between performance-avoidance goals and achievement.

### Performance-avoidance goals: a deleterious form of motivation

Performance-avoidance goals are one of the core constructs in achievement goal theory (Dweck, 1986; Nicholls, 1984; see Senko, 2016 for a recent review). This framework differentiates between two kinds of goals that individuals may adopt in learning settings, namely mastery and performance goals. While mastery goals emphasize a genuine desire to develop one’s skills, performance goals refer to the desire to perform well in comparison with others. Scholars also suggested differentiating achievement goals as function of their approach and avoidance valence (Elliot, 1999). This distinction appeared to be especially relevant regarding performance goals, which were divided into performance-approach goals (i.e. trying outperforming others) next to the performance-avoidance goals (i.e. trying not performing worse than others).

Performance-avoidance goals were consistently shown to have negative consequences on many achievement-related outcomes as well as on achievement itself. Endorsing them was found to be associated with a decrease in intrinsic motivation, task interest, deep processing, and learning efficacy (e.g., Elliot and Church, 1997; Elliot and McGregor, 2001; Huang, 2011, 2016; Hulleman et al. 2010; Liem et al. 2008), an increase in surface processing, test anxiety, disorganization in learning and exam worry (e.g., Elliot and McGregor, 2001; Elliot et al. 2011; Tanaka et al. 2006), perception of tasks as threatening (Elliot and McGregor, 2001), and higher levels of negative achievement emotions and lower levels of positive ones (Huang, 2011; Pekrun et al. 2006). Pursuing these goals has also been found to decrease feedback seeking (Payne et al. 2007) and to result in compliant forms of conflict regulation between peers (Sommet et al. 2014). Finally, a large body of meta-analytical studies has very consistently substantiated the negative impact that performance-avoidance goals exert on achievement, whether it be on performances on cognitive tasks or on students’ achievement in a real classroom (e.g., Huang, 2012; Hulleman et al. 2010; Murayama and Elliot, 2012; Payne et al. 2007; Senko et al. 2011; Van Yperen et al. 2014; Wirthwein, 2013).

### Taking the reasons behind achievement goals into account: the goal complex approach

Recently, theorists from the field started investigating to what extent the effects of achievement goals depend on the reasons for which individuals pursue them. This research has been mostly fueled by the goal-complex approach (e.g., Elliot and Thrash, 2001; Senko and Tropiano, 2016; Sommet and Elliot, 2017), which defines an achievement goal as an end-state that one strives to attain for some idiosyncratic reasons (e.g., Urdan and Mestas, 2006). A goal-complex is a combination between an achievement goal (the “what”) and some particular reason(s) underlying it (the “why”). It is assumed that different goal-complexes may lead to different outcomes, even when the adopted achievement goal is the same. For instance, research showed that pursuing performance-approach goals for autonomous reasons (e.g., because they are challenging) was associated with learning outcomes more positively than the endorsement of the same goals but for controlled reasons (e.g., to comply with the demands of parents or teachers; Gaudreau, 2012; Vansteenkiste et al. 2010). Studies also showed that mastery goals were indeed positively linked with achievement but only when endorsed for social utility reasons (i.e. to succeed at university) and not for social desirability reasons (i.e. to please one’s teachers; e.g., Dompnier et al. 2009).

However, despite the apparent consensus among researchers that taking into account the reasons is necessary for a better understanding of achievement goals, their relative importance remains still controversial (see Sommet and Elliot, 2017, for a review). For instance, Vansteenkiste et al. (2010) showed that both beneficial and deleterious effects of pursuing performance-approach goals were fully explained once autonomous and controlled reasons were controlled for, suggesting that reasons for goal pursuit may be better predictors for educational outcomes than achievement goals themselves. Sommet and Elliot (2017), on the other hand, argued and showed that both mastery and performance-approach goal complexes—that is specifically the combinations of each goal and its underlying reason—account for unique variances in predicting learning outcomes, beyond the variance explained by goals and reasons, even when treated simultaneously.

Since this research has been conducted exclusively on mastery and performance-approach goals, similar issues regarding performance-avoidance goals remain still unanswered. Researchers have not yet addressed the question whether the findings typically yielded on performance-avoidance goals’ effects are conditional to the reasons for which individuals adopt these goals. There is thus room for speculation whether the traditional negative relationships observed between performance-avoidance goals and educational outcomes such as achievement are due to the “true” effects of these goals, or whether they result from some by-default performance-avoidance goal complexes that are implicitly studied but not explicitly captured in standard scales measuring achievement goals. Importantly, this would mean that the typical deleterious effects of these goals found in the literature could depend on some auxiliary assumptions (McGuire, 1983; Świątkowski and Dompnier, 2017) pertaining to some “by-default” reasons implicitly present in the research into achievement goals. Making overt those assumptions would lead to a more fine-grained understanding of how performance-avoidance goals exert their influence on relevant outcomes. Hopefully, doing so should also shed some new light on how achievement goals in general interplay with their reasons to predict external outcomes.

### The social value approach to achievement goal theory

According to recent research into achievement goal theory (Darnon et al. 2009; Dompnier et al. 2009, 2013, 2015; Smeding et al. 2015), goal pursuit can be better understood by taking into account the characteristics of the social environment where it takes place, here the university system. Darnon et al. (2009; see also Jury et al., 2017) argued that some achievement goals could be valued in academia because of their perceived fit with its functional constraints—in particular the education and selection functions—thus making these goals a suitable means to attain success. Furthermore, some achievement goals could be valued because of their perceived fit with social norms endorsed at university—for instance, the ideology of learning—hence making these goals very popular amongst university teachers. These two kinds of “fit” between achievement goals and structural constraints of university correspond respectively to the two facets of social value, namely social utility and social desirability (Beauvois, 2003; Beauvois and Dubois, 2009). Social utility refers to individuals’ capacity to satisfy the functional requirements of a given social environment and indicates the degree to which they are likely to succeed in this environment. Social desirability refers to individuals’ capacity to satisfy the motivations and expectations of the members of a given social group and illustrates the degree to which they are likely to be appreciated. Darnon et al. (2009) showed that on average, only mastery and performance-approach goals are regarded as socially useful and only mastery and performance-avoidance goals are regarded as socially desirable (at least amongst psychology students). Importantly, this research suggested that students are clearsighted about how achievement goals are valued in academia and know how to use this knowledge to influence teachers’ judgments on each component of social value.

Bearing in mind this reasoning, endorsing achievement goals can be viewed as serving two distinct but not mutually exclusive purposes to reach social value in educational contexts (Dompnier et al. 2009, 2013, 2015). First, students may report pursuing achievement goals because they genuinely believe in their efficiency to succeed at university. This reason would be grounded on the perception that an achievement goal is socially useful and would indicate that one’s endorsement of an achievement goal highly matches a genuine commitment with the goal at hand. Second, students may also report pursuing achievement goals because they know their teachers will appreciate them by doing so. This reason would be based on the perception that an achievement goal is socially desirable and could indicate that one’s endorsement of the goal is contaminated by self-presentation strategies and faked. Endorsing a goal for this reason would more likely reflect a strategy to garner teachers’ approval than a true commitment with the goal. This implies that social utility and social desirability reasons underlying the endorsement of achievement goals can dramatically change their meaning as measured by self-report goal scales. Social utility and social desirability are expected then to exert additive but reverse moderating roles on the effects of achievement goals on external outcome. Prior research has supported these predictions in real classroom settings with respect to mastery goals (Dompnier et al. 2009, 2015; Smeding et al. 2015) and performance-approach goals (Dompnier et al. 2013). In both cases, results showed that the degree to which students perceived these achievement goals as socially useful or socially desirable moderated the predictive validity of their spontaneous endorsement: while the relationship between endorsing the goals and grades was enhanced by the increase of their perceived social utility, it was undermined by the increase of their perceived social desirability. To sum up, taking into account achievement goals’ social value allows “discriminating students who endorse these goals for different reasons, namely for self-presentation purposes (social desirability) or for success purposes (social utility), and enables to quantify a qualitative change in the meaning of participants’ answers to an achievement goal scale” (Dompnier et al. 2013, p. 594).

### Hypotheses and research overview

Considering the ubiquitous competitive climate in academia (Darnon et al. 2009; Jury et al., 2017) some students could believe indeed that seeking to not being outperformed by others is what one should do to succeed and act accordingly to show his or her teachers to have the qualities required to this end. Also, past research pointed out that striving to not being outperformed could be associated with some social value of modesty (Darnon et al. 2009), and students could accordingly put forward their endorsement of performance-avoidance goals to be liked by their teachers. The present research seeks to better understand the impact of endorsing performance-avoidance goals on achievement for such social value reasons, anchored in respectively in social utility and social desirability. We propose two mutually exclusive sets of hypotheses about the moderating role of social value reasons on the relationship between the endorsement of performance-avoidance goals and achievement.

Hypothesis 1 assumes that research consistently documented a negative link between performance-avoidance goals and achievement because students seldom report pursuing them for social utility reasons (see Darnon et al. 2009). This assumption relies on the argument put forward by some scholars according to which the effects of achievement goals are driven by the kind of reasons that support their endorsement (e.g., Vansteenkiste et al., 2009). An intriguing possibility could be that performance-avoidance goal-complex based on social utility reasons could transform the classical negative relationship between these goals and achievement into a positive relationship, because students would pursue them for functional reasons for one’s success. Accordingly, Hypothesis 1 expected performance-avoidance goals’ social utility to moderate positively the link between goal endorsement and achievement: The more students would perceive these goals as being socially useful, the more positive would be the link between their performance-avoidance goal endorsement and their achievement.

Hypothesis 2 assumes that pursuing performance-avoidance goals for social utility reasons is expected to reinforce the commitment with the goals and therefore to exacerbate their genuine effects. It relies on the assumption that goal complexes produce outcomes that result from interactive effects obtained through the very combination between an achievement goal and the underlying reason (see Sommet and Elliot, 2017), given that striving to not being outperformed by others exerts an actual deleterious impact on achievement. Accordingly, it is predicted that performance-avoidance goal-complex based on social utility reasons should reveal the negative relationship between these goals and achievement even more strongly. Hypothesis 2 thus expected performance-avoidance goals’ social utility to moderate negatively the link between endorsing performance-avoidance goals and achievement: The more students would perceive these goals as being socially useful, the more negative would be the link between endorsing performance-avoidance goals and achievement.

Depending on which hypothesis is found to be supported over another has also consequences on our expectations about the moderating role played by social desirability reasons. Pursuing achievement goals for social desirability reasons is assumed to reflect self-presentation strategies rather than a true commitment with the goal (e.g., Dompnier et al. 2009), which reduce the predictive validity of the measurement tool (see Smeding et al. 2017). Since Hypothesis 1 assumes that it is social utility that drives positively the effects of pursuing performance-avoidance goals on achievement, we should expect accordingly social desirability reasons to moderate the link between endorsing these goals and achievement in the reverse, negative direction (Corollary of Hypothesis 1). Conversely, since Hypothesis 2 assumes that social utility exacerbates the genuine negative effects of endorsing these goals on achievement, we should expect accordingly social desirability reasons to moderate the relationship between pursuing performance-avoidance goals and achievement in the reverse, positive direction (Corollary of Hypothesis 2).

The aim of Study 1 was to decide between Hypothesis 1 and Hypothesis 2 and their respective corollaries. Across five samples of students from three different countries (United States, Switzerland and France), we assessed performance-avoidance goals’ social utility and desirability as continuous individual difference variables. In order to achieve the maximum level of statistical power, we used a meta-analytic procedure to analyze the findings obtained in these samples pooled altogether. Study 2 aimed at confirming correlational results obtained in Study 2 by experimentally manipulating performance-avoidance goals’ social utility. In both studies, performance-approach goals were also systematically measured for control purposes. Raw data and Supplementary material used in this research are available through OSF website (https://osf.io/8w6tc/).

## Study 1

### Method

#### Samples

Details about the samples are summarized in Table 1. Overall, participants were 638 students from various university departments (e.g., STEM, social sciences, arts) at different academic levels (from 1st year students to graduate students), from the United States, Switzerland and France. The number of participants for Samples 1 and 2 were determined to conveniently detect the effect size of performance goals’ effects on achievement based on Murayama and Elliot (2012; Study 3): we almost tripled the sample size of the original study (Simonsohn, 2015). Samples sizes in Samples 3, 4 and 5 were limited by the number of students in university classes.^1^ Finally, whereas Sample 1 was composed exclusively by native English speakers, Samples 2 to 5 included only native French speakers. Accordingly, the performance goals scale was used in the original English version in Sample 1, whereas it was translated by our own means into French for Samples 2 to 5. The material presented to the participants was thus adapted to fit with their respective linguistic abilities. Sample 1 data were collected online, Sample 2 data were collected in a lab and Samples 3 to 5 data were collected in classroom settings. Such variability in study design was incorporated on purpose in this research to be representative of the usual literature on achievement goals. Finally, participants took part in this research voluntarily and were debriefed at the end of each study.

**Table 1:** Overview of the samples, tasks and measures (samples 1–5)

	*N*	% of Females	Population	Mean age *(SD)*	Task
1	152	36	American	25.4 (6.6)	Modular arithmetics
2	150	56	Swiss	21.6 (2.4)	Anagrams
3	89	77	Swiss	21.9 (1.7)	Exam
4	107	82	Swiss	21.1 (1.9)	Exam
5	140	84	French	19.5 (3.2)	Exam

***Sample 1.*** One hundred and fifty-two American college students from various university sections were recruited online via the Prolific platform. They were told they would take part in a study on cognitive performance and working memory among college students. Participants first completed the practice set of modular arithmetic problems (baseline measure of achievement). They then answered the questionnaire assessing the extent to which they endorsed performance goals during the task, as well as their beliefs about the social value of these goals. Finally, participants performed the second set of modular arithmetic problems (main measure of achievement).

***Sample 2.*** One hundred and fifty students were individually recruited on the university campus and were told that they would participate in a lab study on cognitive performance and working memory.^2^ All were French-speaking Swiss students from various sections of social sciences and humanities and some were from STEM sections. They first completed the practice set of anagrams (baseline measure of achievement). Upon the completion of this set, participants filled up the questionnaire. Finally, they performed the test set of anagrams (main measure of achievement).

***Sample 3.*** Data were collected during a semester-long course in social psychology, involving 115 French-speaking Swiss students in social sciences. Only 89 participants for whom all relevant measures could be retrieved retained in the final sample. At the beginning of the semester, participants answered the questionnaire measuring performance goals and their two components of social value. Therein, they provided an estimation of their academic level during their last year of studies on a seven-point scale (*1* = *very low; 7* = *very high*), which served for controlling initial differences in achievement. The final exam score was retrieved from the class teacher at the end of the semester and served as the main measure of achievement.

***Sample 4.*** Data were collected during a two semester-long course in social psychology class, involving 334 French-speaking Swiss students in social sciences. Only participants for whom all relevant measures could be retrieved were retained in the final sample, which comprised 107 students.^3^ At the end of semester 1, participants answered the questionnaire. During the same week, participants passed their final semester 1 exam, which was used to control for initial difference in achievement level. At the end of the academic year, students passed their final semester 2 exam, which served as the measure of achievement.

***Sample 5.*** Data were collected during a semester-long course in social psychology, involving 165 French students in psychology. We retained in the final sample 140 participants for whom all relevant measures could be retrieved. At the beginning of the semester, participants answered the questionnaire. Therein, they reported their grades relative to the baccalaureate (the final exam passed at the end of high school in France), which served for controlling the initial differences in achievement. The final exam score was retrieved from the class teacher at the end of the semester and served as the main measure of achievement.

#### Procedure

Throughout all the samples, participants’ spontaneous endorsement of performance-avoidance goals was measured with reference to the task or to the class, as well as their beliefs about these goals’ social utility and social desirability. As in Dompnier et al. (2009), the self-presentation paradigm was used for this purpose (Gilibert and Cambon, 2003). This procedure involves answering the scale three times with different instructions: first with “standard” instructions and then according to two within-participants conditions, namely “social utility” and “social desirability” instructions. Under the “standard” instructions, participants were simply asked to indicate their personal level of agreement with each of the three items. Under the “social utility” instructions, they had to respond to the items as if they were to demonstrate that they had all the qualities required to succeed at University in the eyes of their teachers. Under the “social desirability” instructions, they were asked to respond to the scale as if they had to demonstrate that they possessed all the qualities required to please their teachers. In order to measure uncontaminated scores of a priori endorsement of performance goals, the standard instructions are always presented first. The presentation order of “social utility” and “social desirability” instructions was then counterbalanced across participants.^4^

#### Measures

##### Performance-avoidance goals

were assessed with the AGQ-R scale (Elliot and Murayama, 2008) using 3 items (e.g., “I try to avoid doing worse than others”).

##### Performance-approach goals

were assessed for control purposes with the same scale, using 3 items (e.g., “It is important to me to perform better than the other students”).

##### Achievement

In Sample 1, achievement was assessed with a modular arithmetic task (Beilock et al. 2004; see online Supplementary Material). It involved solving two sets of 30 modular arithmetic problems for three minutes for each set. The score on this task was computed by summing the number of correctly solved problems. In Sample 2, an anagram task in French was developed to serve as a proxy of achievement based on the one used in Murayama and Elliot (2012, Study 3; see online Supplementary Material). This task involved solving two sets of 16, five-letter, single-solution anagrams for five minutes for each set. The score on this task was calculated by summing the number of correctly solved anagrams. In both of the tasks, the first set is a practice set used to control for individual prior differences in achievement (baseline achievement), whereas the second set is a test set used as the measure of achievement. In Samples 3, 4 and 5, students’ grades on a university class exam served as a measure of achievement (e.g., Dompnier et al. 2009, 2013). In Samples 3 and 4, the exam grade corresponded to the sum of correct answers on a true/false 50-items questionnaire on the course content, converted into a score ranging from 0 to 6, as typical in the Swiss educational system. In Sample 5, the exam was a multiple-choice questionnaire including 30 questions on the course content, presented each with four possible answers. Points were subtracted in case of incorrect answers. The grade exam corresponded to the sum of correct answers minus incorrect answers that was converted into a score ranging from 0 to 20, as typical in the French educational system. Likewise, Samples 3, 4 and 5 included a baseline measure of achievement serving for controlling the initial differences in achievement level. In Sample 3, it was students’ estimation of their academic level during their last year of studies. In Sample 4, it was students’ grades from semester 1 exam, similar to semester 2 exam. In Sample 5, it was students’ grades from the baccalaureate, ranging from 0 to 20 with 10 as the pass value.

### Data analytic strategy

Throughout all the five samples, data were analyzed using the same regression model involving 17 predictors. Students’ achievement was regressed on performance-avoidance goals, social utility and social desirability of these goals, participants’ baseline level of achievement and all the interaction products between these terms. Participants’ baseline level of achievement was included to ensure that any observed relationship of interest would not be an artifact due to their prior differences in ability. Finally, performance-approach goals and their interaction product with performance-avoidance goals were also controlled, since both goals are correlated (see Muryama and Elliot, 2012) and to account for the unique variance resulting from pursuing multiple goals (see Barron and Harackiewicz, 2001). Before the analyses, all measures (both predictors and dependent variables) were standardized within each sample. To estimate the two effects addressing Hypotheses 1 and 2 and their respective corollaries, random-effects meta-analyses (Borenstein et al., 2009; Hedges and Vevea, 1998) were performed on beta slopes obtained from the regression model estimated in each sample. These analyses were performed in R (R Core Team, 2015) using *metafor* package (Viechtbauer, 2010).

### Results

Descriptive statistics are summarized in Table 2. Correlation matrices for each sample are included in “Appendix 2”. The meta-analytical estimations for each beta slope from the model are included in the Table 3.

**Table 2:** Means, standard deviations and scale reliability coefficients for continuous variables (study 1)

Sample	Performance-avoidance goals			Performance-avoidance goals’ social utility			Performance-avoidance goals’ social desirability			Performance-approach goals			Baseline achievement		Achievement	
	Mean (SD)	Range	*α*	Mean (SD)	Range	*α*	Mean (SD)	Range	*α*	Mean (SD)	Range	*α*	Mean *(SD)*	Range	Mean *(SD)*	Range
1	6.1 (2.7)	1–10	.97	5.6 (2.8)	1–10	.95	5.8 (2.7)	1–10	.94	7.3 (2.4)	1–10	.94	16.9 (7.0)	0–30	19.4 (6.1)	0–30
2	3.7 (1.5)	1–7	.92	4.0 (1.7)	1–7	.84	3.8 (1.6)	1–7	.89	3.8 (1.6)	1–7	.88	7.0 (3.6)	0–16	8.2 (3.4)	0–16
3	3.0 (1.4)	1–7	.90	3.0 (1.7)	1–7	.87	2.7 (1.5)	1–7	.90	3.2 (1.4)	1–7	.91	4.7 (0.9)	1–7	5.0 (0.5)	1–6
4	3.5 (1.7)	1–7	.93	3.4 (1.8)	1–7	.91	3.1 (1.7)	1–7	.92	3.4 (1.6)	1–7	.91	4.8 (0.5)	1–6	4.8 (0.4)	1–6
5	3.4 (1.5)	1–7	.90	3.7 (1.8)	1–7	.83	3.5 (1.6)	1–7	.90	3.7 (1.5)	1–7	.85	12.9 (1.9)	0–20	9.3 (3.8)	0–20

**Table 3:** Random-effects meta-analyses of beta slopes from the model estimated in Study 1

Predictor	*ß*	*95% CI*	*Z*	*T*^*2*^	*I*^*2*^	*Q*
Baseline	.45	[.25, .66]	4.34***	.04	84.5%	28.54***
PAV	.04	[–.07, .14]	0.69	.00	20.2%	3.99
PAP	.03	[–.06, .12]	0.72	.00	8.2%	5.05
UPAV	–.04	[–.13, .03]	–1.15	.00	0.0%	1.10
DPAV	–.09	[–.18, –.01]	–2.19*	.00	5.0%	4.13
Baseline * PAV	–.03	[–.12, .05]	–0.81	.00	0.0%	5.83
Baseline * UPAV	–.03	[–.12, .05]	–0.78	.00	0.0%	2.25
Baseline * DPAV	.07	[–.01, .15]	1.61	.00	0.0%	0.41
PAV * UPAV	–.09	[–.18, –.01]	–2.24*	.00	0.0%	4.70
PAV * DPAV	.03	[–.05, .12]	0.84	.00	0.0%	4.03
UPAV * DPAV	.00	[–.07, .08]	0.12	.00	0.0%	0.20
PAV * PAP	.02	[–.04, .09]	0.68	.00	2.2%	5.20
Baseline * PAV * UPAV	.12	[.03, .20]	2.77**	.00	0.0%	1.33
Baseline * PAV * DPAV	–.05	[–.12, .03]	–1.14	.00	0.0%	3.43
Baseline * UPAV * DPAV	–.03	[–.14, .08]	–0.55	.01	42.0%	7.59
PAV * UPAV * DPAV	.02	[–.04, .09]	0.71	.00	0.0%	1.25
Baseline * PAV * UPAV * DPAV	–.02	[–.08, .04]	–0.66	.00	0.0%	1.73

PAP, Performance-approach goals; PAV, Performance-avoidance goals; *UPAV,* Social Utility of Performance-avoidance goals; DPAV, Social Desirability of Performance-avoidance goals; Baseline, Baseline level of achievement; *95% CI,* Lower and upper limits of 95% confidence interval; *z* test for significance of *ß*; *T*^*2*^, between-studies variance; *I*^*2*^, Percentage of the total variability reflecting real differences in ß*i*; *Q*, homogeneity estimate

^***^ *p* < .001, ** *p* < .01, * p < .05, ^†^ *p* < .10

**Fig. 1:**
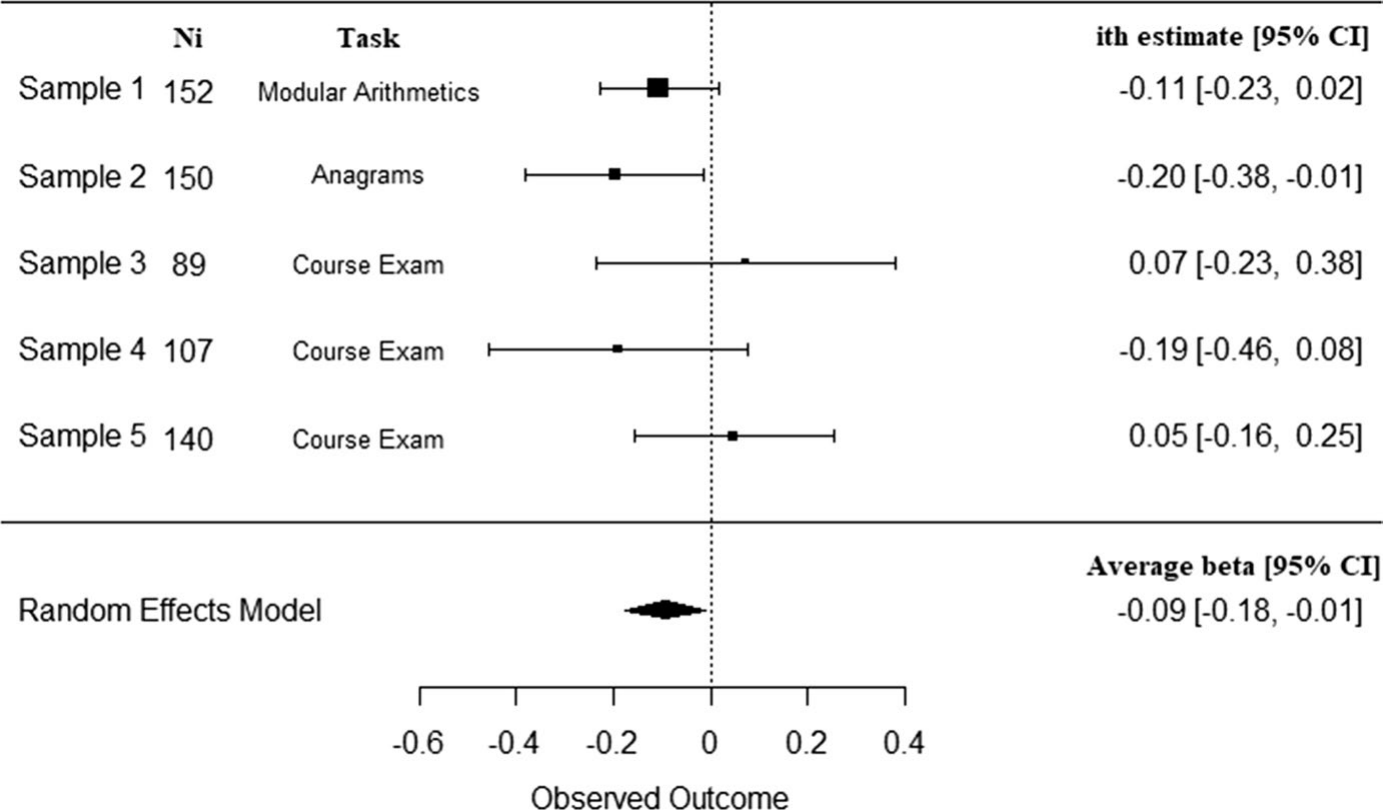
Meta-analytical relationship between achievement and performance-avoidance goal endorsement as a function of these goals’ perceived social utility

**Fig. 2:**
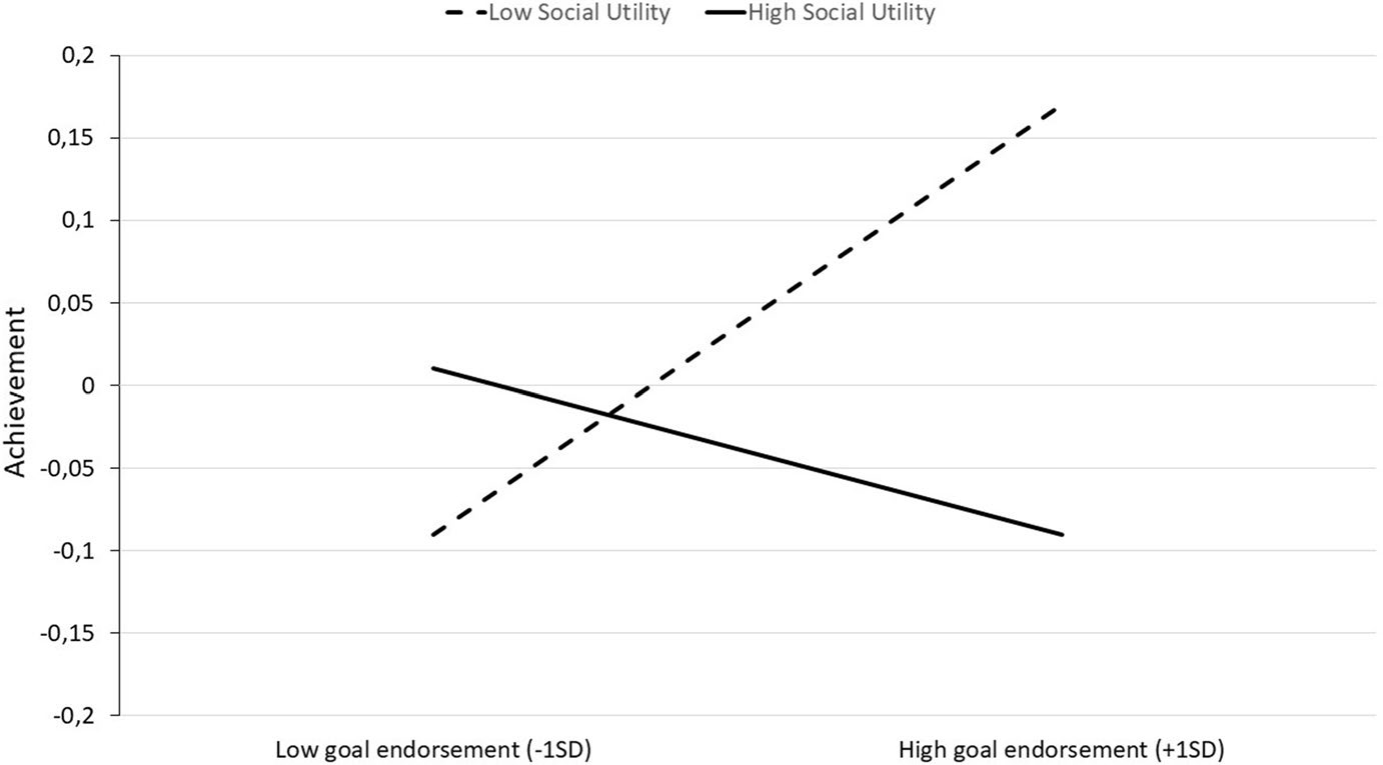
Meta-analytic relationship between performance-avoidance goals and achievement at low (–1SD) and at high (+ 1SD) levels of these goals’ social utility (Study 1)

The meta-analysis relative to the moderation of the link between the endorsement of performance-avoidance goals’ and achievement by social utility of these yielded a negative estimate, *ß* = -0.09, *Z* = -2.24, *p* < 0.02*,* 95% CI = [-0.18, -0.01] (see Fig. 1). No heterogeneity was significantly detected, *Q*(4) = 4.70, *p* = 0.32, and no variability in true effect sizes was observed beyond random sampling error, I^2^ = 0.0%. Thus, as predicted by Hypothesis 2, the higher the students perceived performance-avoidance goals as a mean to succeed, the more negative the relationship between these goals’ endorsement and achievement. Figure 2 presents simple slopes corresponding to this moderation to illustrate the meta-analytic relationship between performance-avoidance goals and achievement as a function of social utility of these goals.

A similar analysis performed on the moderation effect by social desirability of performance-avoidance yielded a positive estimate, as predicted by Corollary of Hypothesis 2. However, this effect was not significant, *ß* = 0.03, *Z* = 0.83, *p* = 0.40, 95% CI = [-0.05, 0.12]. No heterogeneity was significantly detected, *Q*(4) = 4.03, *p* = 0.40, and no variability in true effect sizes was observed, I^2^ = 0.0%.

### Discussion

Study 1 offers substantial evidence enabling to decide between our two competing hypotheses about the role played by social utility: Hypothesis 2 was supported by the data whereas Hypothesis 1 was not. Over and above variations in terms of student populations or performance assessment methods, data indicate that reasons anchored in social utility negatively moderate the link between performance-avoidance goals and achievement. As far as the moderation effect of social desirability is concerned, the meta-analytic estimate of this effect was not statistically significant. We speculate that this could be explained by a lower magnitude of the effect compared to the moderation effect by social utility. Second, it is also possible that the participants in the first two samples (an online and lab studies) could have been less motivated to gain social approval from their teachers than those in other samples, who answered the questionnaire during a real academic course.^5^ Such a difference could produce an overall underestimation in the effect size.

To sum up, Study 1 provided evidence in favor of Hypothesis 2. It remains that such findings are limited by the fact that these studies used correlational designs and rely upon a measure of students’ idiosyncratic beliefs about performance-avoidance goals’ social value. This precludes from drawing any conclusion about the causal role that places social value on the link between performance-avoidance goals and achievement. This limitation was addressed in Study 2 in which performance-avoidance goals’ social utility was experimentally manipulated. Study 2 had a twofold objective: To replicate Study 1′s meta-analytical findings and to test the causal role played by social utility on altering the link between performance-avoidance goals endorsement and achievement.

## Study 2

### Method

#### Participants

One hundred and fifty-six students from two French-speaking Swiss universities took part in this study on a voluntary basis. One participant appeared to be an outlier in the tested model (owing to a value of -3.37 on uncommon standardized residuals) and was discarded from the analyses. The final sample comprised 155 participants, including 91 female and 64 male students (*M*_*age*_ = 22.5, *SD* = 3.1). To determine the sample size, we followed the same rule as Study 1 for Samples 1 and 2, namely we tripled the sample size of the original study (Murayama and Elliot, 2012, Study 3) to detect the effect size of performance goals’ effects on achievement.^6^

#### Procedure

The design of this study was based on Dompnier et al. (2015, Study 1), with the exception that we used the anagram task described above. The first step of the study consisted in measuring participants’ baseline level of performance on the task. The second step consisted in the experimental manipulation of performance-avoidance goals’ social value. Hence, after performing the first set of anagrams, participants were asked to perform an interim task (see Dompnier et al. 2015). Depending on the experimental condition, participants read a bogus scientific article manipulating the level of performance-avoidance goals’ social utility and maintaining constant their level of social desirability (see online Supplementary Material). In one condition, the text included a paragraph presenting research showing that endorsing performance-avoidance goals was positively associated with achievement at University (high-social utility) whereas in the other condition the endorsement of these goals was described as being unrelated with academic achievement (neutral-social utility). In addition, in both conditions, the bogus article presented in another paragraph research showing that performance-avoidance goals did not influence university teachers’ judgments (neutral social desirability). The presentation order of the two paragraphs was counterbalanced across participants. After participants read the bogus article, they were asked to complete a judgment task that was designed to measure the effectiveness of the experimental manipulation of performance-avoidance goals’ social utility (see “Appendix 1”). After completing this task, participants reported their level of endorsement of performance-avoidance (*α* = *0.92*) and performance-approach goals (*α* = 0.90) during the practice set with the AGQ-R scale. Finally, they performed the second set of anagrams. Participants were then thanked and debriefed.

### Results

Results from the manipulation check confirmed the effectiveness of the experimental manipulation (see “Appendix 1”). Descriptive statistics and correlations between all continuous variables are summarized in “Appendix 3”.

Data were analyzed using a regression model in which students’ final performance was regressed on nine predictors: The two experimental conditions—coded using a contrast opposing the “neutral-social utility” condition (coded -0.5) against the “high-social utility” condition (coded 0.5) –, participants’ baseline level of performance (score on the first set of anagrams), their score of endorsement of performance-avoidance goals and all interaction products between these terms. As in Study 1, performance-approach goals and their interaction with performance-avoidance goals were included for control purposes. All continuous predictors were centered on their means prior to the analysis. Summary of the regression analysis can be found in Table 4.

**Table 4:** Output from the regression analysis (Study 2)

Predictor	*ß* estimate	*t*-value	*p*-value	*95% CI*	*η*^*2*^_*p*_
Baseline	.55***	9.03	< .001	[.43, .67]	.36
PAV goals	–.12	–0.73	.47	[–.47, .22]	.00
PAP goals	.35^†^	1.81	.07	[–.03, .73]	.02
CONT	–.04	–0.12	.90	[–.82, .73]	.00
PAP goals * PAV goals	.16^†^	1.66	.09	[–.03, .36]	.02
Baseline * PAV goals	–.09^†^	–1.88	.06	[–.18, .00]	.02
Baseline * CONT	.21^†^	1.75	.08	[–.03, .45]	.02
PAV Goals * CONT	–.71*	–2.51	.02	[–1.27, –.15]	.04
Baseline * PAV Goals * CONT	–.10	–1.15	.25	[–.27, .07]	.01

“Baseline”, Baseline level of achievement; “PAV”, Performance-avoidance; “PAP”, Performance approach goals; “CONT” Contrast coding for the experimental manipulation; *95% CI*, Lower and upper limits of 95% confidence interval; *η*^*2*^_*p*_*, Partial eta square*

^***^ *p* < .0001, ** *p* < .01, * p < .05, ^†^ *p* < .10

**Fig. 3:**
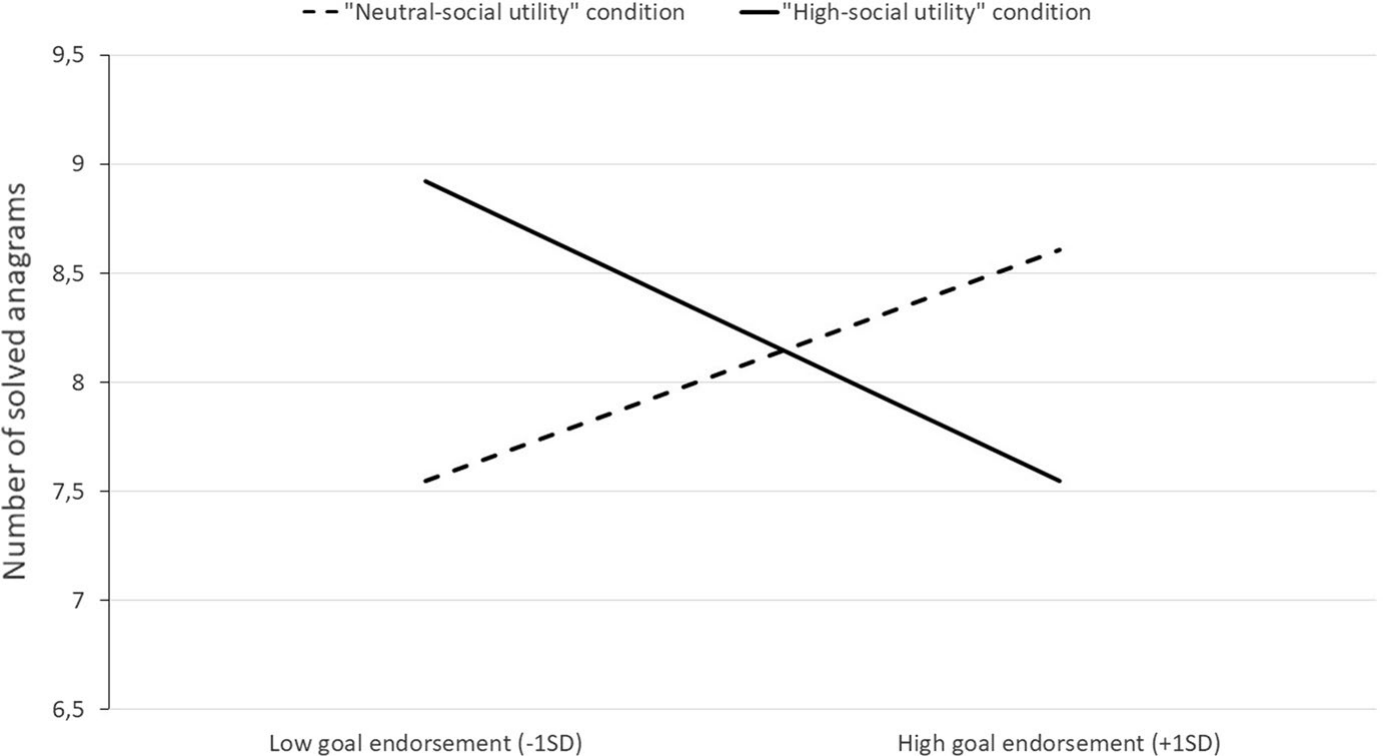
Relationship between final performance and performance-avoidance goal endorsement as a function of these goals’ manipulated social utility (Study 2)

In line with Hypothesis 2, the analysis yielded a negative interaction between the endorsement of performance-avoidance goals and the experimental manipulation of these goals’ social utility, *b* = -0.71, *t*(145) = -2.51*, p* < 0.02, 95% CI = [-1.27, -0.15], η^2^_p_ = 0.04.^7^ Performance-avoidance goals had a more negative relationship with final performance in the “high-social utility” condition than in the “neutral-social utility” condition (see Fig. 3). Specifically, analysis of simple slopes indicated that in the latter condition, endorsing performance-avoidance goals had a positive but not statistically significant effect on participants’ final performance, *b* = 0.23, *t*(145) = 1.07*, p* = 0.29, whereas in the former condition this effect was negative, *b* = -0.48, *t*(145) = -2.06*, p* < 0.05.

### Discussion

This study was conducted to replicate Study 1′s meta-analytical findings and to test the causal role played by social utility on the link between the endorsement of performance-avoidance goals and performance using an experimental design. Results obtained confirmed those obtained in Study 1: Social utility associated with performance-avoidance goals negatively moderated the link between these goals and final performance in the direction, as predicted by Hypothesis 2. When performance-avoidance goals were described as being highly socially useful, their endorsement had a more negative impact on participants’ performance than when these goals were presented as being neutral on social utility. Hence Study 2 replicates this effect but also strengthens its internal validity. The use of experimental design allows concluding that the very change in students’ perception of performance-avoidance goals’ social utility is indeed responsible for altering the relationship between the spontaneous endorsement of these goals and the performance on a cognitive task.

## General discussion

A consistent body of research has documented the negative link between endorsing performance-avoidance goals and achievement. The present research sought to investigate whether this relationship is conditional to the reasons underlying the pursuit of these goals. Five correlational samples (Study 1) involving students from three different countries and one experiment (Study 2) were conducted to test two mutually exclusive hypotheses on the direction of the moderation of the link between performance-avoidance goals and achievement by social value reasons.

Results from both studies provided a clear support for Hypothesis 2, which assumed that the negative effect of performance-avoidance goals on achievement is due to their genuine effect, which was exacerbated by social utility reasons. Study 1 demonstrated that performance-avoidance goals’ perceived social utility increased the negative link between their spontaneous endorsement and students’ achievement, over and above population and achievement measurement differences across the samples. Furthermore, Study 2 demonstrated the causal role played by social utility in altering the relationship between performance-avoidance goals and achievement in the direction predicted by Hypothesis 2. Though the Corollary of Hypothesis 2 was not supported in the present research regarding the moderation of performance-avoidance goals’ effect on achievement by social desirability, we believe that additional studies replicating this research in more appropriate settings (i.e. in real university classes) could bring more conclusive evidence.

### Limitations

This work is also limited in several aspects. Firstly, the three different countries used in Study 1 from which we drew the samples (U.S., Switzerland and France) were selected by convenience. We did not expect to observe any notable differences across the samples and indeed no heterogeneity was detected when testing for our hypotheses. Still, additional research is needed to assess whether systematic cross-cultural variations exist between those countries with respect to how performance-avoidance goals predict achievement. Secondly, note that Study 2 was limited by the absence of an a priori power analysis justifying the sample size. However, the level of achieved power (0.71) indicates that it was underpowered only to a minor extent considering the usual reference level of 0.80. Finally, despite the fact that Study 1 provided evidence in line with Hypothesis 2, it failed to support the corollary of this hypothesis, that is the positive moderating role of social desirability. As outlined above, such lack of conclusive evidence could indicate that some contexts, which would motivate students to obtain social approval from their teachers (e.g., real academic courses), would be more appropriate to test this specific effect. Future research should address this possibility by investigating this moderation in such contexts more thoroughly.

### Conclusions

To the best of our knowledge, this is the first empirical research supporting the idea that the effects of performance-avoidance goals on achievement are dependent on the reasons underlying the endorsement of these goals. It extends previous research into achievement goals based on the social value approach. The evidence presented here is consistent with past research on mastery (Dompnier et al. 2009) and performance-approach goals (Dompnier et al. 2013), showing that social utility reasons exacerbate in reverse direction the predictive validity of performance-avoidance goals on achievement. Hopefully, these results will bring some food for thought to the current debate regarding the importance of reasons underlying goal pursuit in explaining learning outcomes. Our results support the relevance of studying achievement goal complexes as motivational constructs that offer a more in-depth picture of achievement motivation than achievement goals and reasons for goal pursuit considered alone (see Sommet and Elliot, 2017). The current findings indicate that is the very combination between performance-avoidance goals and the underlying specific reasons that accounts the best for individuals’ achievement. Even more importantly, they showed that pursuing these goals for seemingly good, competence-relevant reasons—in order to succeed (i.e. social utility reasons)—lead to negative consequences on achievement. Such results are especially compelling because past research showed that pursuing mastery or performance-approach goals for social utility reasons increase the positive effects of pursuing these goals. This means that pursuing different goals for the same reasons can lead to literally opposite consequences, which highlights the utility of studying achievement goal complexes.

At the practical level, these results may present some implications. Data from Study 1 indicate that more than half of students who reported striving to not perform worse than their peers to a high extent pursed such goals because they believe in their efficiency as a means to succeed at academia.^8^ Past research showed that individuals who are the most inclined to adopt the performance-avoidance goals are low-achievers (Senko and Harackiewicz, 2005) and first-generation students (Jury et al. 2015). Despite the fact that these goals are not by default particularly valued at university, some of these students could still happen to believe in their efficiency as a means to succeed. This could be especially true with regards to the first-generation students who, at the beginning of their curriculum, may still not be fully aware of the academic culture, norms and values (Autin et al. 2015). Consequently, these students could pursue performance-avoidance goals for seemingly good reasons—in order to succeed—that could still actually have a counter-productive effect on their level of achievement. As a matter of fact, such a rationale falls in line with the results obtained in Study 1. As visible in Table 3, the lower the baseline level of achievement, the more negative the interactive effect between performance-avoidance goals and their social utility on achievement is. Consistently with past research on mastery goals (Dompnier et al. 2015), it appears that low-achievers are those who are particularly prone to rely on their knowledge of social value of performance-avoidance goals and see their achievement deteriorate consequently. Therefore, educators who would wish to discourage their students from endorsing performance-avoidance goals should also insist that these goals could be deleterious even if endorsed for “right” reasons.

In the light of the present evidence, performance-avoidance goals may appear as an inherently deleterious form of motivational regulation, since even pursuing them for good reasons seems to lead to a decrease in performance. We consider such a bold conclusion to be premature. Previous research has pointed out some contexts where performance-avoidance goals can be in fact beneficial for performance, for instance under stereotype threat (Chalabaev et al., 2012, 2014), when associated with a dominant prevention focus (Świątkowski and Dompnier, 2020), or among students from Asian cultures (Hulleman et al. 2010; King, 2016). We thus believe there is still much yet to learn and understand about how exactly performance-avoidance impact individuals’ achievement. The studies presented here rather support the idea that social utility related reasons function as a stimulating factor that allows monitoring personal commitment with an achievement goal. Therefore, one could reasonably expect that endorsing performance-avoidance goals for social utility reasons to give rise to beneficial consequences, had they been endorsed in social or cultural contexts that may be supportive for their positive effect. Future studies should focus on identifying such contexts to better understand in which conditions performance-avoidance goals are unequivocally deleterious for achievement and when they are not. For instance, seeking to not underperform in comparison with one’s peers may be beneficial for one’s learning in some Asian populations because such a motivation fits their collectivist self-construal (King, 2016). A plausible expectation would be then that endorsing these goals in such a collectivist context for social utility reasons could have a positive effect on learning outcomes. In other words, pursuing performance-avoidance goals in this context because one is convinced about their usefulness to reach success would make their positive effect even stronger. Examining such an intriguing possibility constitutes an interesting avenue for prospective research.

## Appendix

### Manipulation check task (study 2).

Participants were asked to put themselves in the shoes of a university teacher and judge a fictitious student based on his high endorsement of performance-avoidance goals on a performance-avoidance goal scale. This scale included three items extracted from the revised version of achievement goals (Elliot and Murayama, 2008). The target showed a high level of agreement with each of these three items: The scores 6, 5 and 6 were encircled on a 7-point scale ranging from 1 (*not at all*) to 7 (*very much*). Participants were asked to describe the fictitious students on a set of six personality traits pertaining to social desirability (e.g., likeable, pleasant, sympathetic, α = 0.83) and five personality traits referring to social utility (e.g., competent, intelligent, gifted, α = 0.76). This judgment task was presented as a filler task but was actually a manipulation check of the experimental manipulation. After completing this task, participants answered the same three performance-avoidance goal items on a seven-point scale (1 = *not at all for me*; 7 = *very true for me*) under standard instructions (α = 0.92) in order to assess their spontaneous endorsement of these goals.

We tested whether the experimental manipulation through the use of a bogus article successfully altered the participants’ perception of performance-avoidance goals’ social utility and social desirability. An independent sample Student t-test comparing participants’ judgment scores on social utility in accordance with their experimental condition (high vs. neutral social utility) revealed the expected effect of social utility manipulation, *t*(152) = 2.36, *p* < 0.02, 95% CI = [0.05, 0.58], η^2^ = 0.04, indicating that the target was judged as being more socially useful in the high-utility condition (*M* = 4.59, *SD* = 0.93) than in the neutral-social utility condition (*M* = 4.27, *SD* = 0.72). This result indicates that the experimental manipulation affected participants’ beliefs about performance-avoidance goals’ social utility in the expected direction. A similar analysis comparing participants’ judgment scores on social desirability as function of their experimental condition yielded no statistically significant effect of social utility manipulation, *t*(152) = -1.06, *p* = 0.29, 95% CI = [− 0.37, 0.11].

### Correlation matrices for each sample (study 1).

Tables 5, 6, 7, 8, 9.

**Table 5:** Correlation matrix for sample 1

	1	2	3	4	5	6
1. Social utility of performance-avoidance goals	–					
2. Social desirability of performance-avoidance goals	.50***	–				
3. Performance-avoidance goal endorsement	.39***	.39***	–			
4. Performance-approach goals endorsement	.07	.16*	.35***	–		
5. Baseline achievement	–.16*	–.08	–.07	.27***	–	
6. Final achievement	–.22**	–.18*	–.14^†^	.25***	.78***	–

*** *p* < .001, ** *p* < .01, ** p* < .05, ^†^ *p* < .10

**Table 6:** Correlation matrix for sample 2

	1	2	3	4	5	6
1. Social utility of performance-avoidance goals	–					
2. Social desirability of performance-avoidance goals	.47***	–				
3. Performance-avoidance goal endorsement	.25***	.27***	–			
4. Performance-approach goals endorsement	.11	.15^†^	.64***	–		
5. Baseline achievement	.04	–.07	.03	.15^†^	–	
6. Final achievement	–.02	–.19*	–.04	.00	.57***	–

*** *p* < .001, ** *p* < .01, ** p* < .05, ^†^ *p* < .10

**Table 7:** Correlation matrix for sample 3

	1	2	3	4	5	6
1. Social utility of performance-avoidance goals	–					
2. Social desirability of performance-avoidance goals	.16	–				
3. Performance-avoidance goal endorsement	.31***	.24	–			
4. Performance-approach goals endorsement	.16	.04	.56***	–		
5. Baseline achievement	.00	–.08	.04	.26**	–	
6. Final achievement	–.02	.03	.17	.23*	.32***	–

*** *p* < .001, ** *p* < .01, ** p* < .05, ^†^ *p* < .10

**Table 8:** Correlation matrix for sample 4

	1	2	3	4	5	6
1. Social utility of performance-avoidance goals	–					
2. Social desirability of performance-avoidance goals	.55***	–				
3. Performance-avoidance goal endorsement	.25**	.30***	–			
4. Performance-approach goals endorsement	.15	.17 ^†^	.68***	–		
5. Baseline achievement	–.09	.00	–.07	.11	–	
6. Final achievement	–.01	–.05	.05	.03	.30***	–

*** *p* < .001, ** *p* < .01, ** p* < .05, ^†^ *p* < .10

**Table 9:** Correlation matrix for sample 5

	1	2	3	4	5	6
1. Social utility of performance-avoidance goals	–					
2. Social desirability of performance-avoidance goals	.45***	–				
3. Performance-avoidance goal endorsement	.26***	.25***	–			
4. Performance-approach goals endorsement	.02	.08	.47***	–		
5. Baseline achievement	–.07	–.15 ^†^	.08	.20*	–	
6. Final achievement	–.10	–.11	.19*	.28***	.40***	–

*** *p* < .001, ** *p* < .01, ** p* < .05, ^†^ *p* < .10

### Means, standard deviations and correlations among continuous variables (study 2)

**Table:**

			Correlations			
Measure	M	SD	1	2	3	4
1. Performance-avoidance goals endorsement	3.63	1.42	–			
2. Performance-avoidance goals endorsement	3.9	1.40	0.54***	–		
3. Baseline performance	7.66	3.40	–0.10	0.15	–	
4. Final performance	8.35	3.05	–0.04	0.17*	0.59*	–

*** *p* < 0.001, ** p* < 0.05.

## Footnotes

1 Samples 2, 3, 4 and 5 from Study 1 are part of a larger project (Świątkowski and Dompnier 2020). They included measures that are not relevant for the present research and are not reported.

2 This study was a conceptual replication of Murayama and Elliot (2012, Study 3). It involved a manipulation of structural competition. Participants were led to think they would complete an anagram task in co-action settings but in reality, performed the study alone. Some were told that they would perform the second anagram task in competition with a participant, and some were told to perform the task they best they could. This manipulation is neither relevant for the purposes of this research, nor any interactive effect between this variable and other predictors of interest in the main analyses has been detected. Hence, we do not develop on this further.

3 The high drop rate in Sample 4 was due to the fact that students enrolled to the course for semester 1 were not required to enroll for semester 2. Out of the initial 334 participants, only 107 were enrolled and actually passed the exams for both semesters.

4 In each study, performance-approach goals were assessed using the same procedure, resulting in three measures: spontaneous endorsement, social utility and social desirability of these goals. The last two measures are not reported since they are not relevant for the present research.

5 Sample-by-sample analysis revealed that this moderation effect was found in Sample 4, *ß* = .33, *t*(88) = 2.45*, p* < .02, 95% CI = [.06, .62], η^2^_p_ = .06, meaning that a real university class might be a more appropriate setting to detect this effect.

6 Note that an a priori power analysis was not possible, since no past research has investigated the interaction effect of social utility on the link between endorsing performance-avoidance goals and achievement using an experimental design.

7 The inclusion of the outlier into the dataset does not change the statistical conclusion of the analysis: *b* = -.60, *t*(146) = -2.05*, p* < .05, 95% CI = [-1.17, -.02], η^2^_p_ = .03.

8 Out of 638 students from Study 1, 255 scored above the mid-point on performance-avoidance goals scale. Out of these 255 students, 144 (56%) scored above the mid-point on performance-avoidance goals’ social utility.
